# Effect of DL-Methionine Supplementation on Tissue and Plasma Antioxidant Status and Concentrations of Oxidation Products of Cholesterol and Phytosterols in Heat-Processed Thigh Muscle of Broilers

**DOI:** 10.3390/ani10112050

**Published:** 2020-11-05

**Authors:** Johanna O. Zeitz, Tamara Ehbrecht, Anne Fleischmann, Erika Most, Denise K. Gessner, Silvia Friedrichs, Marion Sparenberg, Klaus Failing, Rose Whelan, Dieter Lütjohann, Klaus Eder

**Affiliations:** 1 Institute of Animal Nutrition and Nutritional Physiology, Justus Liebig University, D-35392 Giessen, Germany; Johanna.O.Zeitz@ernaehrung.uni-giessen.de (J.O.Z.); Tamara.Ehbrecht@vetmed.uni-giessen.de (T.E.); Anne.Fleischmann@vetmed.uni-giessen.de (A.F.); Erika.Most@ernaehrung.uni-giessen.de (E.M.); Denise.Gessner@ernaehrung.uni-giessen.de (D.K.G.); 2 Institute of Clinical Chemistry and Clinical Pharmacology, University Hospital Bonn, D-53127 Bonn, Germany; Silvia.Friedrichs@ukbonn.de (S.F.); Dieter.Luetjohann@ukbonn.de (D.L.); 3 Unit of Biomathematics and Data Processing, Faculty of Veterinary Medicine, Justus Liebig University, D-35392 Giessen, Germany; Marion.Sparenberg@vetmed.uni-giessen.de (M.S.); Klaus.Failing@vetmed.uni-giessen.de (K.F.); 4 Evonik Operations GmbH, D-63457 Hanau-Wolfgang, Germany; rose.whelan@evonik.com

**Keywords:** antioxidants, broiler, cholesterol oxidation products, methionine, phytosterol oxidation products

## Abstract

**Simple Summary:**

The occurrence of oxidative stress is a general problem in high-yielding farm animals. Oxidative stress not only affects animal health, but free radicals generated under oxidative stress conditions can also promote the oxidation of lipids in animal products which are detrimental for the consumer, such as lipid oxidation products. Therefore, prevention of oxidative stress by improving the antioxidant system has high priority in farm animal management. Methionine (Met) is a precursor of glutathione, one of the major antioxidants in the body. Therefore, we investigated the hypothesis that feeding diets with an excess of Met in relation to the requirement for optimum growth improves the antioxidant status by enhancing the formation of glutathione in broilers. We observed that broilers fed diets with an excess of Met had higher concentrations of glutathione in liver and thigh muscle, indicative of an improved antioxidant status, and moreover had lower concentrations of cholesterol oxidation products in heat-processed thigh muscle, in comparison to broilers of the control group. This effect is favorable with respect to animal health and quality of broiler meat.

**Abstract:**

In this study, the hypothesis that supplementation with methionine (Met) as DL-Met (DLM) in excess of the National Research Council (NRC) recommendations improves the antioxidant system in broilers was investigated. Day-old male Cobb-500 broilers (n = 72) were divided into three groups which were fed a control diet or diets supplemented with two levels of DLM in which the concentrations of Met + Cys exceeded the recommendations of NRC by 15–20% (group DLM 1) or 30–40% (group DLM 2), respectively. The three groups of broilers did not show differences in body weight gains, feed intake, and feed conversion ratio. However, broilers of groups DLM 1 and DLM 2 had higher concentrations of glutathione (GSH) in liver and thigh muscle and lower concentrations of cholesterol oxidation products (COPs) in heat-processed thigh muscle than broilers of the control group. Concentrations of several oxidation products of phytosterols in heat-processed thigh muscle were also reduced in groups DLM 1 and DLM 2; however, the concentration of total oxidation products of phytosterols was not different between the three groups. The study shows that DLM supplementation improved the antioxidant status due to an increased formation of GSH and reduced the formation of COPs during heat-processing in thigh muscle.

## 1. Introduction

Supplementing methionine (Met) to broiler diets in order to balance the amino acids in accordance to the broilers demand is a common practice. However, Met has several import functions apart from its role as a proteinogenic amino acid [1]. For instance, S-adenosyl-Met—a metabolite of Met—is acting as a precursor of homocysteine, from which cysteine (Cys) is formed via the trans-sulfuration pathway [2]. Cys is moreover required for the formation of taurine and glutathione (GSH), both of which have important functions as antioxidants [3]. Dietary supplementation of Met could therefore improve the antioxidant defense system of the body via increased concentrations of these Met metabolites. According to the role of Met in the antioxidant system, it has been shown that an insufficient supply of dietary-sulfur-containing amino acids (SAA) attenuates the antioxidant system in broilers [4,5]. The aim of present study is to investigate the hypothesis that supplementation of Met in excess of the requirement enhances the antioxidant system in broilers. It is well-known that some commonly used parameters of lipid peroxidation, such as concentrations of thiobarbituric acid-reactive substances (TBARS), are relatively unspecific parameters of lipid peroxidation [6]. In contrast to TBARS, oxidation products of cholesterol (cholesterol oxidation products, COPs) and phytosterols (phytosterol oxidation products, POPs) are much more specific indicators of lipid oxidation [7,8]. Moreover, COPs and POPs are relevant with respect to human health as they promote the development of several diseases including coronary heart disease or cancer [9,10]. COPs and POPs are formed predominately during heat-processing of foods containing cholesterol or phytosterols, respectively [11,12,13,14]. In order to investigate the hypothesis that supplementation of Met in broilers reduces the formation of COPs and POPs, we determined the concentrations of individual COPs and POPs in heat-processed thigh muscle of broilers. 

## 2. Materials and Methods 

### 2.1. Animals, Husbandry, Diets, and Experimental Design

Seventy-two day-old male broiler chickens (Cobb 500, Cobb, Wiedemar, Germany) were divided into three experimental groups, with 24 broilers each, which were kept in pens with eight broilers per pen (3 pens per group). The pens were 0.11 m above the ground, and their dimensions were 2.05 m × 1.05 m × 0.86 m (length/width/height). The floor of the pens was equipped with cardboards whose upper crinkled cardboard layers were exchanged 2 times per week (day 1–14) or every 2 days (days 14–35). The pens were randomly distributed within the room. They contained nipple drinkers and automated feeders allowing the birds free access to feed and water. Light intensity and the light:dark regime were in accordance with our recent study [15]. The room temperature decreased from 29.5 °C on day 1, measured at pen height, to 21.0 °C on day 35. During the first 6 days, infrared lamps were used as additional heat sources. Mean relative humidity was 60.0 ± 1.9%. The control group received basal diets in a three-phase feeding system (starter diet from day 1 to day 10, grower diet from day 11 to day 21, finisher diet from day 22 to day 35). The composition of the three basal diets are shown in Table 1. During the first 2 days, the diets were provided in a crumbled form. From day 3 to day 45, the diets were provided in pellet form, with a diameter of 2 mm. No vitamin E was supplemented, in order to avoid masking of the effects of methionine on the antioxidant system. The basal diets met or even exceeded the broiler’s requirements of nutrients and amino acids according to the breeder’s recommendations [16]. The concentration of Met + Cys in the basal diet was in agreement with the recommendations of the National Research Council (NRC) [17] for broiler diets. However, the concentrations of standardized ileal digestible (SID) Met + Cys in the diets were about 15–20% below the recommendations of the breeder and about 10% lower than those recommended by AMINOChick 2.0 (Evonik Nutrition & Care GmbH, Hanau-Wolfgang, Germany). The treatment groups received the basal diets supplemented with DL-methionine (DLM) at two different concentrations (Table 2). In the groups whose diets were supplemented with the lower (DLM 1) or the higher (DLM 2) concentration of DLM, concentrations of Met + Cys exceeded recommendations of NRC [17] for Met + Cys by 15–20% and 30–40%, respectively. The concentrations of SID Met + Cys in the DLM 1 diets were close to the recommendations of the breeder and those of AMINOChick 2.0. The concentration of SID Met + Cys in the DLM 2 diets exceeded the recommendations of the breeder by 15–20% and those of AMINOChick 2.0 by 13–19%. The animal experiment was approved by the Animal Welfare Officer of the Justus Liebig University, (Giessen, Germany), JLU No. 637_M.

### 2.2. Sample Collection and Laboratory Analysis

Sample collection and laboratory analyses were largely according to our recent study [15]. On day 35, the birds were killed by bleeding (opening of Vena jugularis and Arteria carotis) after an electrical anesthesia. Twelve birds per group (4 birds from each pen) whose body weights represented the means of the whole group were selected for sample collection. Weights of carcass, breast muscle, thighs (including bones), and liver of these birds were recorded. Whole blood was collected into 10 mL tubes containing ethylenediaminetetraacetic acid as an anticoagulant. Plasma was prepared by centrifugation and stored at −20 °C. Samples of liver and thigh muscle were snap-frozen in liquid nitrogen and stored at −80 °C. The right thigh (including skin and bones) was collected and stored at −20 °C for later heat-processing. For heat-processing, the frozen thigh was thawed overnight. The thawed thighs were heated in a drying oven (T6200, Thermo Fisher Scientific, Osterode, Germany), at a temperature of 170 °C for 50 min. Afterwards, the thighs were cooled at ambient temperature. The skin was removed, and a sample of the muscle (M. puboischiofemoralis) was taken, placed into a 2 mL vial, snap frozen with liquid nitrogen, and then stored at −80 °C pending analysis.

Dry matter (DM) and crude nutrients (crude protein, ether extract, crude fiber, and crude ash) in feed samples were determined by the official methods of Verband der Deutschen Landwirtschaftlichen und Forschungsanstalten (VDLUFA) [19]. Amino acids in feed samples were analyzed by ion-exchange chromatography [20,21]. Vitamin C was analyzed by HPLC-ECD [22]. The concentrations of α- and γ-tocopherol were determined by HPLC [23]. The concentrations of TBARS were determined by fluorescence spectrometry [5]. The concentrations of phytosterols, COPs (7α-hydroxycholesterol (7α-OH cholesterol), 7β-hydroxycholesterol (7β-OH cholesterol), 7-ketocholesterol) and POPs (7α-hydroxychampesterol (7α-OH champesterol), and 7β-hydroxycampesterol (7β-OH champesterol), 7-ketocampesterol, 7α-hydroxysitosterol (7α-OH sitosterol), 7β-hydroxysitosterol (7β-OH sitosterol), and 7-ketositosterol) in heat-processed thigh muscle were determined by gas chromatography–mass spectrometry [24,25]. Cholesterol was determined by gas chromatography–flame ionization detection [26]. For technical reasons, analyses of cholesterol, phytosterols, COPs, and POPs could be performed only in 8 samples per group which were randomly selected. The concentrations of GSH in blood and tissues were determined by HPLC-MS [27,28]. More details of the analytical procedures are described by Zeitz et al. [15].

### 2.3. Statistical Analysis

Statistical evaluation of the data was performed by the Minitab statistical software (Rel. 13.1, Minitab, State College, PA, USA). All the data were tested for normal distribution. If the distribution of a variable was skewed to the right, the data were transferred to their logarithms before statistical analysis, and data are shown as geometric mean with its dispersion factor (=geometric standard deviation). The effects of the treatment were evaluated by analysis of variance (ONEWAY). If F values were statistically significant, the means of the three groups were compared by Tukey test. Effects were considered significant at *p* ≤ 0.05. Correlation analysis was carried out with the linear regression tool from Minitab. 

## 3. Results

### 3.1. Effect of DLM Supplementation on Growth Performance of Broilers

Supplementation of DLM did not influence performance data (body weight gains, final body weight, feed intake, and feed:gain ratio) and carcass characteristics (carcass weight; dressing percentage; and percentages of breast muscle, thighs, and liver) of the broilers (Table 3).

### 3.2. Effect of DLM Supplementation on Concentrations of Antioxidants and TBARS in Plasma and Tissues of Broilers

The groups supplemented with DLM (groups DLM 1 and DLM 2) had higher concentrations of GSH in the liver and in thigh muscle than the control group; the concentration of GSH in blood did not differ between the three groups of broilers (Table 4). Concentrations of other antioxidants (vitamin C and total tocopherols) and oxidation products (TBARS) in plasma, liver, and thigh muscle showed largely no differences between the groups supplemented with DLM and the control group (Table 4). The only exception of this was an increase of vitamin C in thigh muscle of broilers of group DLM 1 in comparison to the control group (Table 4).

### 3.3. Effect of DLM Supplementation on Concentrations of Cholesterol, Phytosterols, and Their Oxidation Products in Heat-Processed Thigh Muscle of Broilers

The concentration of cholesterol in heat-processed thigh muscle did not differ between the three groups of broilers (Table 5). Broilers of groups DLM 1 and DLM 2 had lower concentrations of 7α-OH cholesterol, 7β-OH cholesterol, and 7-ketocholesterol, and the sum of these COPs in heat-processed thigh muscle was lower than in control broilers, both per g of tissues and per mol of cholesterol (Table 5). Concentrations of all the phytosterols in heat-processed thigh muscle were not different between the three groups of broilers (Table 6). However, broilers of groups DLM 1 and DLM 2 had lower concentrations of 7β-OH campesterol, 7α-OH sitosterol and 7β-OH sitosterol than the control group (Table 6). Group DLM 1 moreover had a lower concentration of 7α-OH campesterol and group DLM 2 had a lower concentration of 7-ketocampesterol than the control group (Table 6). The concentration of 7-ketositosterol did not differ between the three groups of broilers. Broilers of groups DLM 1 and DLM 2 had a lower concentration of total oxidized campesterols than the control group (Table 6). The concentration of total oxidized sitosterols and the concentration of total oxidized phytosterols (oxidized campesterols + oxidized sitosterols) were not different between the three groups of broilers. Concentrations of all the oxidized campesterols and oxidized sitosterols expressed per mol of campesterol or sitosterol, respectively, were also lower in groups DLM 1 and DLM 2 than in the control group, with the only exception of the concentration of 7-ketositosterol, which was not different between the three groups (Table 6).

### 3.4. Correlations between Concentrations of Antioxidants in Plasma and Tissues and the Concentrations of COPs and POPs in Heat-Processed Thigh Muscle of Broilers 

There were no significant correlations between the concentrations of antioxidants (vitamin C, tocopherols, and GSH) in blood, plasma, and liver and the concentrations of COPs and POPs in heat-processed thigh muscle (data not shown). However, there were significant inverse correlations between the GSH concentration in thigh muscle and the concentrations of total and individual COPs (7α-OH cholesterol, 7β-OH cholesterol, and 7-ketocholesterol) in heat-processed thigh muscle (Table 7). Moreover, there were significant inverse correlations between the vitamin C concentration and the concentrations of total and individual COPs (7α-OH cholesterol, 7β-OH cholesterol, and 7-ketocholesterol), total oxidized campesterols, and total POPs in heat-processed thigh muscle (Table 7).

## 4. Discussions

This study investigated the effect of dietary DLM supplementation on the antioxidant system in broilers. In contrast to our recent study, which investigated the effect of Met supplementation to a diet in which the Met concentration was 15% below NRC requirements [5], we used basal diets which were still below those recommended by the breeder but met requirements according to NRC [17]. The finding that animal performance in the present study did not differ between the three groups of broilers implies that the Met + Cys concentration in the basal diets was already sufficient for maximum growth under the specific conditions in this experiment. However, it should be noted that the small number of replications for assessment of growth performance (n = 3) does not yield results with a sufficient statistical power. Therefore, the performance data should be interpreted carefully. Moreover, it should be noted that the effect of the treatment on growth performance was not the primary aim of this study.

The data of this study show that dietary Met concentrations which are 15–20% above the breeder´s recommendations, compared to diets 15–20% below those recommendations, have only minor effects on the concentrations of tocopherols and vitamin C in the plasma, liver, and thigh muscle of broilers. The only exception of this was an increase of the concentration of vitamin C in thigh muscle of the broilers of group DLM 1. As concentrations of antioxidants such as tocopherols or vitamin C are expected to be lowered under condition of oxidative stress due to an increased consumption by reaction with ROS [29], it is expected that a reduction of oxidative stress would lead to increased concentrations of these antioxidants. Thus, these results would indicate that supplementation of DLM in slight excess of the recommendations has limited effects on the antioxidant system. However, we observed a significant increase of GSH in liver and thigh muscle of broilers supplemented with DLM. This observation might be due to the fact that GSH forms a storage pool of readily available Cys, which is increased when Met availability increases [2]. Previous studies have already shown that supplementation of Met increases the formation of GSH in liver and thigh muscle of broilers [4,5,15,30,31,32]. GSH is one of the major water-soluble antioxidants in the body, and thus an increased GSH concentration could imply that the antioxidant status was improved by DLM supplementation. In contrast to the suggestion of an increased antioxidant status of broilers supplemented with DLM, concentrations of TBARS in plasma, liver, and thigh muscle remained unchanged by DLM supplementation. The concentrations of TBARS are often determined in studies dealing with oxidative stress as an indicator of lipid peroxidation. However, it should be noted that TBARS represent a relatively unspecific parameter of lipid peroxidation due to the fact that thiobarbituric acid reacts with a variety of aldehydes and breakdown products of proteins and carbohydrates [6]. Thus, the observation that TBARS concentrations were largely unaffected by the treatment must not necessarily be contradictory to the suggestion that supplementation of DLM caused an improvement of the antioxidant status.

Cholesterol, a natural compound in animal tissues located mainly in cell membranes, is susceptible to oxidation, which results in the formation of COPs. The formation of COPs in animal tissues is strongly increased by heat-processing. It has been observed that concentrations of COPs in breast or thigh muscle of broilers are increased 10- to 40-fold by heating at a temperature of 180 °C for 20 min [11]. The formation of COPs is not only a valid indicator of the susceptibility of a tissue towards lipid peroxidation, but COPs are also relevant from a nutritional point of view, as their intake in humans is associated with the risk of various diseases, such as cardiovascular disease or cancer [10]. We observed that supplementation of DLM led to a reduction of the concentrations of individual and total COPs in heat-processed thigh muscle. This finding agrees with a previous study which has shown that supplementation of DLM reduces the formation of COPs in heat-processed thigh muscle in broilers subjected to heat stress [15]. The inverse correlations between the concentration of GSH and vitamin C in thigh muscle and the concentrations of COPs in heat-processed thigh muscle indicates that the reduced formation of COPs was due to increased concentrations of those antioxidants in the muscle. The concentrations of total COPs in heat-processed thigh, being in the range between 10 and 30 nmol/g sample in the three treatment groups, were in the same order of magnitude as those detected in cooked broiler meat considered in other studies [33,34]. Although the concentrations of COPs found in heated thigh muscle are much lower than in, e.g., dried egg products, heat-processed meat products contribute considerably to the intake of COPs in consumers [35]. Thus, the reduction of the concentration of COPs by supplementation of DLM might be regarded as a favorable effect, not only with respect to animal health, but also with respect to product quality of broiler meat.

In this study, we also determined the concentrations of phytosterols and POPs in heat-processed thigh muscle. Phytosterols are very similar to cholesterol with respect to their structures and functions [36]. We were able to detect several phytosterols in thigh muscle of broilers, with campesterol, sitosterol, and campestanol as the major components. The concentration of total phytosterols, being around 150 nmol/g (equivalent to 5.8 mg/100 g) in heat-processed thigh muscle of the control broilers, is much lower than in most plant foods, such as in cereals (50–100 mg/100 g [37]) or vegetable oils (150–600 mg/100 g [38]). Thus, broiler meat will contribute less to the total daily intake of phytosterols in Western diets which is commonly around 200 to 400 mg at a daily energy intake of 2000 kcal [39,40,41]. Phytosterols are regarded as beneficial compounds in human nutrition, as they are able to reduce the absorption of cholesterol in the intestine, although significant effects on plasma cholesterol concentration are only reached by supplementation of 1.5 to 3 g [42,43]. Like cholesterol, phytosterols are susceptible to autoxidation, with 7α-hydroxy-, 7β-hydroxy-, and 7-keto derivatives being the main oxidation products [36]. In the present study, we were able to detect oxidation products of campesterol and sitosterol in heat-processed thigh muscle. Among the POPs identified, 7-keto derivatives of campesterol and sitosterol showed the greatest concentrations in heated broiler thigh muscle. This agrees with observations that 7-keto derivatives are the most abundant oxidation products of phytosterols in various products [14]. In our study, around 7–8 mol% of sitosterol and 1.2–1.5 mol% of campesterol in thigh muscle were oxidized during heat-processing. These data show that the phytosterols in the broiler thigh muscle were highly susceptible to oxidation during heat-processing. We observed that the oxidation of campesterol to 7α-OH campesterol, 7ß-campesterol, and 7-keto campesterol in heat-processed broiler thigh muscle was reduced by DLM supplementation. Correlation analysis revealed an inverse relationship between the concentration of vitamin C in thigh muscle and the concentrations of POPs in heat-processed thigh muscle, indicating that the slightly increased concentrations of vitamin C in muscle of broilers supplemented with DLM in comparison the control broilers could be responsible for a reduced formation of POPs. Although POPs are poorly absorbed in the small intestine, they are suggested, like COPs, to be atherogenic, and therefore their intake via foods requires attention [9,44]. However, it has to be noted the concentrations of total POPs in heat-processed thigh muscle being in the range between 3.5 and 4 pmol per g (equivalent to 0.875 to 1.00 µg/100 g) of thigh muscle are much lower than in other foods, such as plant oils (0.5 to 7 mg/100 g in different samples of corn oil or sunflower oil), or products baked with margarines, such as cookies or muffins (0.2–0.7 mg/100 g) [13,45]. Thus, the consumption of heat-processed broiler meat is uncritical with respect to the ingestion of POPs.

## 5. Conclusions

This study shows that supplementation of DLM in excess of the requirement for Met + Cys recommended by NRC [17] has only minor effects on the concentrations of tocopherols and vitamin C but causes an increase of the concentration of GSH in the liver and thigh muscle, and thus overall improves the antioxidant system in broilers. In agreement with the indication of an increased antioxidant status, the formation of COPs in heat-processed thigh muscle was reduced by DLM supplementation. This effect is favorable with respect to animal health and quality of broiler meat.

## Figures and Tables

**Table 1:** Compositions of the basal diets.

Item	Starter Diet	Grower Diet	Finisher Diet
Component (%)			
Corn	50.0	54.9	58.8
Soybean meal	30.6	29.2	26.0
Linseed oil	2.76	3.60	4.16
Maize gluten	7.21	7.65	7.00
Fish meal	5.00		
Monocalcium phosphate	1.43	1.53	1.24
Limestone	1.45	1.33	1.12
Mineral and vitamin premix ^#^	1.00	1.00	1.00
Salt	0.24	0.36	0.33
Choline chloride	0.10	0.12	0.13
L-Lysine (54.6%)	0.10	0.19	0.16
L-Threonine (98.5%)	0.05	0.06	0.05
L-Valine (98.0%)	0.03	0.03	0.01
Nutrients (%) ^†^			
DM	89.0 (84.6)	88.9 (85.0)	88.9 (85.3)
Crude ash	7.39 (6.08)	6.85 (6.01)	6.16 (6.01)
Crude fiber	3.03 (2.95)	3.06 (3.12)	2.95 (3.00)
Crude fat	6.09 (5.17)	6.64 (5.71)	7.25 (5.75)
Crude protein	24.9 (23.3)	21.6 (20.4)	20.0 (19.3)
SID Lysine	1.29 (1.22)	1.10 (1.03)	1.00 (0.98)
SID Methionine	0.45 (0.43)	0.38 (0.37)	0.35 (0.34)
SID Methionine + cysteine	0.74 (0.71)	0.65 (0.63)	0.61 (0.59)
SID Threonine	0.82 (0.80)	0.71 (0.69)	0.65 (0.65)
SID Tryptophan	0.23 (–) ^‡^	0.19 (–)	0.18 (–)
Metabolizable energy (MJ/kg)	12.7 (11.8)	13.0 (12.2)	13.3 (12.4)

^#^ The premix contained (per kg): calcium, 300 g; chloride, 1.0 g; retinol, 1,200,000 IU; cholecalciferol, 400,000 IU; menadione, 333 mg; biotin, 25 mg; folic acid, 167 mg; thiamine, 333 mg; riboflavin, 800 mg; pyridoxin, 417 mg; cobalamin, 2.5 mg, nicotinamide, 6.91 g; calcium pantothenate, 2.0 g; choline chloride, 40 g; iron, 5.0 g; copper, 1.5 g; manganese, 10.0 g; zinc, 7.0 g; iodine, 156 mg; selenium, 25 mg. ^†^ Calculated values based on AMINODat^®^ 5.0; analyzed values in brackets. Concentrations of standardized ileal digestible (SID) amino acids were calculated from the analyzed amino acid concentrations with consideration of digestibility values provided by AMINODat^®^ 5.0. Metabolizable energy was calculated based on the concentrations of crude nutrients of the dietary ingredients according to GfE [18]. ^‡^ (–) not determined. DM = Dry Matter.

**Table 2:** Dietary supplementation levels of DLM and concentrations of free Met, Met, and Met + Cys in the diets (%)*.

Diet	DLM ^#^	Met	Met + Cys
Starter			
Control	0 (0.06)	0.50 (0.48)	0.87 (0.83)
DLM 1	0.19 (0.23)	0.69 (0.64)	1.06 (0.99)
DLM 2	0.37 (0.41)	0.87 (0.82)	1.24 (1.15)
Grower			
Control	0 (0.06)	0.42 (0.41)	0.76 (0.73)
DLM 1	0.16 (0.22)	0.58 (0.54)	0.92 (0.86)
DLM 2	0.31 (0.36)	0.73 (0.68)	1.07 (1.00)
Finisher			
Control	0 (0.06)	0.39 (0.38)	0.71 (0.69)
DLM 1	0.15 (0.21)	0.54 (0.51)	0.86 (0.81)
DLM 2	0.29 (0.34)	0.68 (0.64)	1.00 (0.96)

* Abbreviations: Met, methionine; Met + Cys, methionine plus cysteine. Calculated values based on AMINODat^®^ 5.0; analyzed values in brackets. ^#^ Supplementation levels of DLM; analyzed concentrations of free Met (including free Met from supplementation and free Met from dietary ingredients) in brackets.

**Table 3:** Performance data and carcass characteristics of broilers fed a control diet (control) or diets supplemented with two different levels of DL-methionine (DLM 1 and DLM 2) *^,‡,†^.

Item	Control	DLM 1	DLM 2	*p*
Performance data				
Body weight, day 1 (g)	40.5 ± 0.3	40.7 ± 0.6	40.8 ± 0.8	0.736
Body weight, day 45 (g)	2587 ± 16	2551 + 88	2512 + 94	0.515
Weight gain (g)	2546 ± 17	2510 ± 88	2471 ± 95	0.519
Feed intake (g)	3787 ± 73	3720 ± 52	3703 ± 158	0.613
Feed: gain ratio (g/g)	1.48 ± 0.04	1.48 ± 0.04	1.50 ± 0.01	0.765
Carcass characteristics				
Eviscerated carcass weight (g)	1911 ± 100	1900 ± 106	1858 ± 70.8	0.354
Dressing percentage (%)	73.3 ± 1.4	73.6 ± 2.5	73.4 ± 1.8	0.924
Breast muscle (% of body weight)	22.7 ± 1.6	22.2 ± 1.1	22.3 ± 1.6	0.641
Thighs (% of body weight)	19.1 ± 0.7	20.5 ± 1.7	19.8 ± 0.8	0.020
Liver (% of body weight)	1.97 ± 0.20	1.96 ± 0.25	1.86 ± 0.13	0.310

* Results are means ± SD, n = 3 for performance data, n = 12 for carcass characteristics data. ^‡^ DLM 1: 15–20% in excess of NRC [17] requirement; DLM 2: 30–40% in excess of NRC requirement [17]. ^†^ In all the items considered, there were no significant differences between the three groups.

**Table 4:** Concentrations of antioxidants and oxidation products in plasma, blood, liver, and thigh muscle of broilers fed a control diet or diets supplemented with two different levels of DL-methionine (DLM 1 and DLM 2) *^,‡^.

Item ^#^	Control	DLM 1	DLM 2	*p*
Plasma				
Vitamin C (µmol/L)	65.1 ± 11.8	63.2 ± 6.5	57.2 ± 6.0	0.116
Total tocopherols (µmol/L) ^§^	11.5 ± 1.2	11.4 ± 1.8	12.6 ± 2.4	0.248
TBARS (mmol) ^#^	1.49 ± 0.36	1.47 ± 0.52	1.23 ± 0.23	0.224
Blood				
GSH (mmol/L)	2.81 ± 0.30	2.83 ± 0.31	2.98 ± 0.42	0.438
Liver				
Vitamin C (µmol/g)	1.92 ± 0.16	1.99 ± 0.25	1.75 ± 0.16	0.050
Total tocopherols (nmol/g) ^§^	19.7 ± 2.8	19.7 ± 2.5	22.1 ± 3.4	0.152
GSH (µmol/g)	2.61 ± 0.49 ^b^	3.51 ± 0.31 ^a^	3.28 ± 0.61 ^a^	0.002
TBARS (nmol/g)	41.6 ± 8.2	38.8 ± 7.3	40.8 ± 8.3	0.855
Thigh muscle				
Vitamin C (µmol/g)	0.18 ± 0.03 ^a^	0.22 ± 0.02 ^b^	0.19 ± 0.02 ^a^	0.004
Total tocopherols (nmol/g) ^§^	9.97 ± 1.45	8.39 ± 2.00	8.92 ± 2.07	0.075
GSH (µmol/g)	1.13 ± 0.27 ^b^	1.39 ± 0.12 ^a^	1.55 ± 0.16 ^a^	0.002
TBARS (nmol/g) ^†^	5.37; 3.63	5.14; 2.21	4.49; 2.14	0.346

* Results are means ± SD, n = 12. ^‡^ DLM 1: 15–20% in excess of NRC requirement [17]; DLM 2: 30–40% in excess of NRC requirement [17]. ^#^ Abbreviations: GSH, glutathione; TBARS, thiobarbituric acid-reactive substances. ^†^ Geometric means and dispersion factors. ^§^ Total tocopherols represents the sum of α- and γ-tocopherol. ^a, b^ Means without the same superscripts differ significantly (*p* ≤ 0.05).

**Table 5:** Concentrations of cholesterol and cholesterol oxidation products (COPs) in heat-processed thigh muscle of broilers fed a control diet (control) or diets supplemented with two different levels of DL-methionine (DLM 1 and DLM 2) *^,‡^.

Item	Control	DLM 1	DLM 2	*p*
Cholesterol (µmol/g)	10.5 ± 1.14	9.04 ± 0.64	9.53 ± 0.79	0.123
7α-OH cholesterol (nmol/g)	6.86 ± 2.41 ^a^	2.34 ± 0.58 ^b^	3.67 ± 1.56 ^b^	0.001
7β-OH cholesterol (nmol/g)	11.63 ± 4.44 ^a^	4.13 ± 1.04 ^b^	6.81 ± 3.15 ^b^	0.002
7-ketocholesterol (nmol/g)	11.77 ± 4.45 ^a^	4.06 ± 0.85 ^b^	6.19 ± 2.67 ^b^	0.001
Total COPs (nmol/g)	30.3 ± 11.1 ^a^	10.5 ± 2.5 ^b^	16.7 ± 7.4 ^b^	0.001
COPs, relative to cholesterol				
7α-OH cholesterol (mmol/mol)	0.68 ± 0.28 ^a^	0.26 ± 0.08 ^b^	0.41 ± 0.14 ^b^	0.001
7β-OH cholesterol, (mmol/mol)	1.15 ± 0.54 ^a^	0.46 ± 0.14 ^b^	0.77 ± 0.30 ^b^	0.006
7-ketocholesterol, (mmol/mol)	1.16 ± 0.54 ^a^	0.45 ± 0.12 ^b^	0.70 ± 0.25 ^b^	0.003
Total COPs (mmol/mol)	2.99 ± 1.35^a^	1.16 ± 0.34^b^	1.87 ± 0.69^b^	0.002

* Results are means ± SD, n = 8. ^‡^ DLM 1: 15-20% in excess of NRC (National Research Council) requirement [17]; DLM 2: 30–40% in excess of NRC requirement [17]. ^a, b^ Means without the same superscripts differ significantly (*p* < 0.05).

**Table 6:** Concentrations of phytosterols and oxidized phytosterols in heat-processed thigh muscle of broilers fed either a control diet (control) or diets supplemented with two different levels of DL-methionine (DLM 1 and DLM 2) *^,‡^.

Item	Control	DLM 1	DLM 2	*p*
Phytosterols, absolute				
Campesterol (nmol/g)	88 ± 28	90 ± 10	107 ± 43	0.404
Sitosterol (nmol/g)	35 ± 8	34 ± 3	42 ± 19	0.324
Campestanol (nmol/g)	15 ± 4	14 ± 2	17 ± 9	0.374
Stigmasterol (nmol/g)	4.1 ± 0.7	3.7 ± 0.5	5.0 ± 1.8	0.097
Sitostanol (nmol/g)	5.1 ± 0.8	4.6 ± 0.4	6.1 ± 2.6	0.324
Oxidized phytosterols, absolute				
7α-OH campesterol (pmol/g)	0.08 ± 0.03 ^a^	0.04 ± 0.01 ^b^	0.06 ± 0.03 ^a,b^	0.022
7β-OH campesterol (pmol/g)	0.19 ± 0.06 ^a^	0.09 ± 0.02 ^b^	0.13 ± 0.07 ^b^	0.004
7-ketocampesterol (pmol/g)	1.17 ± 0.18 ^a^	1.03 ± 0.10 ^a,b^	0.91 ± 0.18 ^b^	0.013
7α-OH sitosterol (pmol/g)	0.08 ± 0.03 ^a^	0.06 ± 0.01 ^b^	0.05 ± 0.01 ^b^	0.001
7β-OH sitosterol (pmol/g)	0.18 ± 0.04 ^a^	0.13 ± 0.01 ^b^	0.14 ± 0.03 ^b^	0.007
7-ketositosterol (pmol/g)	2.11 ± 0.32	2.37 ± 0.39	2.50 ± 0.60	0.253
Total oxidized campesterols (pmol/g)	1.45 ± 0.23 ^a^	1.13 ± 0.09 ^b^	1.10 ± 0.27 ^b^	0.015
Total oxidized sitosterols (pmol/g)	2.34 ± 0.36	2.56 ± 0.39	2.69 ± 0.62	0.499
Total oxidized phytosterols (pmol/g)	3.76 ± 0.50	3.75 ± 0.45	3.79 ± 0.73	0.927
Oxidized phytosterols, relative to phytosterols *				
7α-OH campesterol (mmol/mol)	1.01 ± 0.40 ^a^	0.48 ± 0.11 ^b^	0.59 ± 0.25 ^b^	0.005
7β-OH campesterol (mmol/mol)	2.32 ± 0.83 ^a^	0.98 ± 0.21 ^b^	1.25 ± 0.52 ^b^	0.001
7-ketocampesterol (mmol/mol)	14.1 ± 3.4 ^a^	11.6 ± 1.9 ^b^	9.5 ± 3.0 ^b^	0.015
7α-OH sitosterol (mmol/mol)	2.34 ± 0.61 ^a^	1.69 ± 0.33 ^b^	1.30 ± 0.47 ^b^	0.002
7β-OH sitosterol (mmol/mol)	5.11 ± 0.97 ^a^	3.86 ± 0.43 ^b^	3.33 ± 0.93 ^b^	0.002
7-ketositosterol (mmol/mol sitosterol)	62.7 ± 12.5	71.2 ± 16.3	55.54 ± 8.9	0.093
Total oxidized campesterols (mmol/mol)	17.4 ± 4.5 ^a^	12.8 ± 2.1 ^b^	11.3 ± 3.5 ^b^	0.008
Total oxidized sitosterols (mmol/mol)	68.4 ± 13.1	76.9 ± 17.2	59.2 ± 9.4	0.088

* Results are means ± SD, n = 8. ‡ DLM 1: 15–20% in excess of NRC (National Research Council) requirement [17]; DLM 2: 30–40% in excess of NRC requirement [17]. * Concentrations of oxidized campesterols are related to campesterol; concentrations of oxidized sitosterols are related to sitosterol. ^a, b^ Means without the same superscripts differ significantly (*p* < 0.05).

**Table 7:** Significant correlations between the concentrations of antioxidants (GSH and vitamin C) in thigh muscle and concentrations of cholesterol oxidation products (COPs) and phytosterol oxidation products (POPs) in heat-processed thigh muscle *.

Variable 1	Variable 2	r	*p*
GSH	Total COPs	−0.57	0.007
	7α-OH cholesterol	−0.62	0.003
	7β-OH cholesterol	−0.51	0.019
	7-ketocholesterol	−0.60	0.004
Vitamin C	Total COPs	−0.57	0.008
	7α-OH cholesterol	−0.56	0.009
	7β-OH cholesterol	−0.57	0.007
	7-ketocholesterol	−0.60	0.004
	Total POPs	−0.43	0.049
	Total oxidized campesterols	−0.44	0.046

* n = 24; r = Pearson’s correlation coefficient. GSH = glutathione.
